# HIV incidence and cohort retention among men who have sex with men in Hangzhou, China: A prospective cohort study

**DOI:** 10.1097/MD.0000000000017419

**Published:** 2019-10-04

**Authors:** Qingchun Li, Xiting Li, Yan Luo, Dai Fang, Junfang Chen, Xingliang Zhang, Xin Lv, Jie Jin, Ke Xu, Wenjie Luo, Han-Zhu Qian

**Affiliations:** a Hangzhou City Center for Disease Control and Prevention, Hangzhou, China; b Department of Biostatistics, Rutgers, The State University of New Jersey, New Brunswick, New Jersey; c Xiacheng District Center for Disease Control and Prevention, Hangzhou, China; d Department of Biostatistics, Yale School of Public Health, New Haven, Connecticut.

**Keywords:** China, human immunodeficiency virus, incidence, prospective cohort study, retention rate, syphilis

## Abstract

Prospective cohort studies have been conducted to estimate HIV incidence among men who have sex with men (MSM) in first-line megacities cities (>10 million residents) in China, but few in the second-line large- or middle-size cities. This study was to investigate HIV incidence and cohort retention among MSM in a second-line city Hangzhou in eastern China.

A total of 523 HIV-seronegative MSM were recruited during September 2014 to September 2015, and were followed up prospectively at 3, 6, 9, and 12 months. Questionnaire interviews were conducted, and laboratory tests were performed to evaluate baseline syphilis infection and HIV seroconversions. Chi-square test and logistic regression model were used to identify factors associated with cohort retention rate and syphilis prevalence.

Of 523 participants, 137 (26.2%) completed 6-month follow-up, and use of Internet for recruiting study participants (vs other recruitments: adjusted odds ratio [AOR] = 0.5; 95% confidence interval [CI]: 0.3–0.8) and being homosexual (vs heterosexual or bisexual: AOR = 0.6; 95% CI: 0.4–0.9) were associated with lower cohort retention. The overall HIV incidence during 12 months of follow-up was 6.6 per 100 person-years (95% CI: 3.4–9.8/100 PY). The prevalence of syphilis at baseline was 6.5% (95% CI: 4.4%–8.6%), and disclosing sexual orientation (AOR = 0.4, 95% CI: 0.2–0.9) was associated with lower risk of syphilis infection.

HIV is spreading rapidly among MSM in the second-line Chinese city. Effective interventions are needed to target this population in both first-line megacities and second-line large and middle-size cities.

## 1 Introduction

Despite progress has been made on preventing HIV transmission, HIV incidence remains unacceptably high among men who have sex with men (MSM) in both high- and low-income countries.^[1]^ In China, MSM represents a group with fastest growth in HIV epidemic. The proportion of newly diagnosed HIV cases who were MSM increased from 12.2% in 2007 to 26.4% in 2016.^[2,3]^ Homosexual contact has become the dominant route of HIV transmission in many parts of this country, particularly in urban areas.^[4–6]^

Prospective cohort studies have been conducted in recent years to measure HIV incidence rate among Chinese MSM, most in the first-line megacities with >10 million urban residents like Beijing, Shanghai, Chongqing, Chengdu, and Shengzhen, which reported HIV incidence ranging from 3.9 to 18.9 per 100 person-years.^[7–10]^ However, few studies have been conducted in second-line large- or middle-size Chinese cities with <10 million population.^[8,11,12]^

Hangzhou is the capital city of Zhejiang Province in eastern China. It is a typical second-line Chinese city with fast-growing economy. As the hometown of Chinese e-commerce giant Alibaba, Hangzhou attracts many young immigrants, including MSM. HIV case reporting data suggested an increasing trend. Cross-sectional studies have shown high prevalence of HIV, syphilis, and unprotected anal intercourse among MSM in Hangzhou.^[13–15]^ A study conducted across Zhejiang province showed that Hangzhou represented the dominant proportion of local HIV transmissions and cross-regional transmissions based on the provincial transmission networks, and possessed the largest number of nodes with ≥50 edges which accounted for 50% of total cases, and Hangzhou City might serve as a central regional role in the intra-provincial spread of HIV.^[16]^ However, there is no HIV incidence data among MSM in Hangzhou. To address this question, we conducted this prospective cohort study among Hangzhou MSM to evaluate HIV incidence; cohort retention; and prevalence of baseline syphilis infection and its associated factors.

## 2 Methods

### 2.1 Study design and participants enrollment

This prospective cohort study was conducted in Hangzhou City, which has 7.23 million local residents and over 2 million migrants, and as a famous tourist city, has >140 million tourists every year. MSM participants were recruited through a gay-friendly community-based organization (CBO) using a variety of approaches including advertisement on gay websites, social media (QQ and WeChat), outreach to MSM-frequented venues, and peer referral. The eligible criteria for participation were: man, age ≥18 years, HIV-seronegative status, self-reported anal or oral sex with another man in the past 3 months, plan to live in Hangzhou for at least 6 months, willing and able to complete procedures, and willing and able to providing written informed consent. Exclusion criteria were HIV-seropositive status and failure to provide contact information. Based on estimated 5% HIV incidence among MSM in Hangzhou, we planned to recruit 400 HIV-negative MSM participants and observe them for 1 year with a follow-up rate of 60% in order to have ±3% precision of incidence estimation. A total of 523 eligible participants were eventually enrolled into the cohort study during September 2014 and September 2015.

Participants were followed up for 12 months. At baseline and every 3 months during follow-up, trained research staff conducted one-on-one questionnaire interviews with participants in a private room. Blood samples were collected from each participant and tested for HIV and syphilis. To ensure confidentiality, each participant was assigned a unique study identification number (PID), which labeled questionnaires and specimens. Each participant was required to provide their PID to receive their test results. Pre- and post-test counseling was provided, along with free condoms and education materials. Each participant was requested to provide 2 contact methods (e.g., cell phone number, email, or WeChat etc), and they were contacted for follow-ups. The study protocol was approved by the institutional review board of Hangzhou Center for Disease Control and Prevention.

### 2.2 Questionnaire interview

The baseline questionnaire included questions on socio-demographic characteristics such as age, ethnicity, sex, education, marital status, residency status, employment, income, sexual orientation, HIV knowledge and awareness, and mental status. The baseline and follow-up questionnaires assessed use of cigarettes, alcohol and illicit drugs, and sexual behaviors, such as number and sex of steady, casual, and commercial sexual partners, condom use, and symptoms of sexually transmitted infections (STIs) in the past 3 months.

### 2.3 Laboratory tests

Blood samples were tested for HIV and syphilis infections in a local health care center which is nearby the CBO. HIV sero-status was screened using ELISA (Wantai BioPharm, Beijing, China) testing, and positive screening results were confirmed using HIV-1/2 Western blot c (MP Biomedicals Asia Pacific Pte. Ltd, Shanghai, China). Syphilis infection status was determined using rapid plasma reagin (RPR) test (Wantai BioPharm, Beijing, China) and *Treponema pallidum* particle assay test (Shanghai Rongsheng Biotech Co. Ltd., Shanghai, China).

### 2.4 Statistical analysis

Questionnaire and laboratory testing results were double entered and checked for accuracy using EpiData software (The EpiData Association Odense, Denmark, version 3.02). Data were analyzed using SAS software (SAS Institute Inc., Cary, NC). The primary study endpoint was HIV seroconversion. HIV seroconversion was estimated to have occurred at the midpoint between the participant's last negative test and first positive test in the 3-month follow-up intervals. HIV incidence density was calculated based on a Poisson distribution, with person-year (PY) over the observed time as the denominator. Chi-square test was used in univariate analyses, variables significant at a level of *P* = .10 were fitted in a multivariate logistic regression model to estimate the factors associated with cohort retention rate and baseline syphilis prevalence. Statistical significance was defined as a 2-sided *P* value <.05. Original data for this paper will be made available by contacting the authors.

## 3 Results

### 3.1 Demographics and sexual behaviors of participants

**Figure 1:**
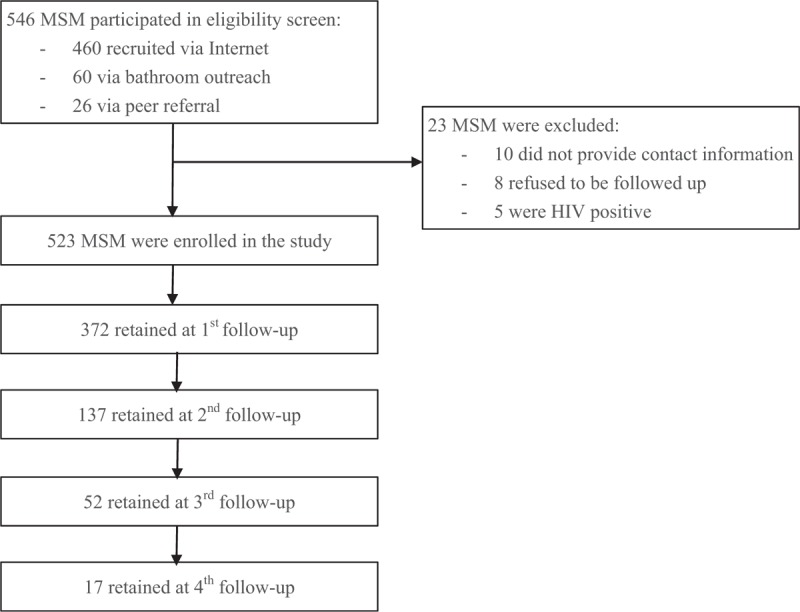
Flow diagram of study participants.

Of 546 MSM who were screened for eligibility, 5 were HIV positive, 10 declined to provide contact information, 8 refused to participate in follow up visits, thus, a total 523 eligible MSM were enrolled into the cohort study (Fig. 1). Of 523 HIV-negative MSM who participated in the prospective cohort study, mean age was 32 years (range, 18–80); about one-third (34%) were married or cohabiting with a female sexual partner, and 36% reported themselves as heterosexuals or bisexuals; nearly one half (47%) were migrants from other provinces; about three quarters (73%) of men had disclosed their sexual orientation (Table 1). Over half (51%) of participants had multiple sexual partners in the past 3 months, but only one-fifth (20%) took >1 HIV test in the past 12 months (Table 1).

**Table 1:**
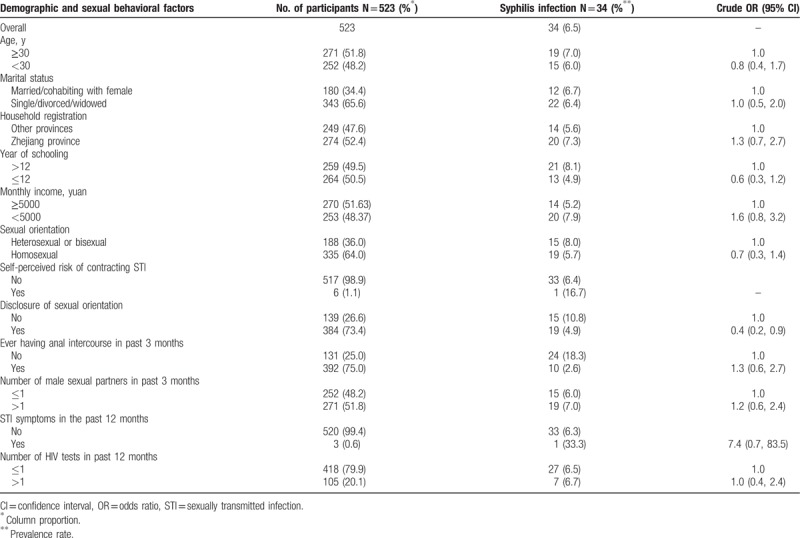
Demographic characteristics and baseline syphilis infection among 523 MSM in Hangzhou, China.

### 3.2 Baseline syphilis prevalence

Of 523 HIV-negative MSM who enrolled in the study, 34 (6.5%, 95% confidence interval [CI]: 4.4–8.6) were syphilis seropositive. Multivariate logistic regression showed that men who disclosed their sexual orientation were 60% less likely to be infected with syphilis than men who did not (adjusted odds ratio [AOR] = 0.4; 95% CI: 0.2–0.9, *P* = .019) (Table 1).

### 3.3 Cohort retention and HIV seroconversion

The cohort retention at 3, 6, 9, and 12 months were 71.1% (372/523), 26.2% (137/523), 9.9% (52/523), and 3.4% (18/523), respectively (Table 2). Multivariate logistic regression analysis showed that men who were recruited through Internet (AOR = 0.5; 95% CI: 0.3–0.8, *P* = .02) and those who reported homosexual orientation (AOR = 0.6; 95% CI: 0.4–0.9, *P* = .04) were less likely retained at 6 months (Table 3).

**Table 2:**
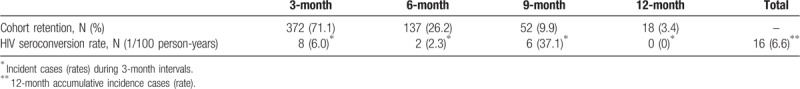
Cohort retention and HIV seroconversion rates during 12 months of follow-up among 523 MSM in Hangzhou, China.

**Table 3:**
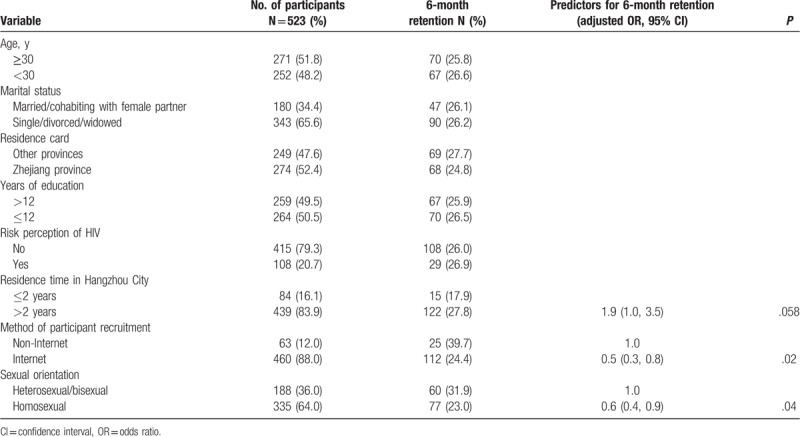
Factors associated with 6-month cohort retention among 523 MSM in Hangzhou, China.

Over the 12-month follow-up period, a total of 16 HIV new seroconversions were detected, and 243.4 person-years (PY) were observed. Therefore, the overall HIV incidence rate was 6.6/100 PY (95% CI: 3.4–9.8/100 PY). The number of HIV seroconversions at the 1st, 2nd, 3rd, and 4th 3-month follow-up intervals was 8, 2, 6 and 0, respectively, which represented HIV incidence rates of 6.0, 2.3, 37.1, and 0.0 per 100 PY (Table 2).

## 4 Discussion

This study showed high incidence of HIV among MSM in the second-line large Chinese city Hangzhou. Previous studies conducted in first-line megacities in China have consistently shown high HIV incidence in this population, for example, 3.9/100 PY (95% CI: 2.4–5.4) to 7.8/100 PY (95% CI: 4.5–12.7) in Beijing,^[6,9,17,18]^ 5.8/100 PY in Guangzhou in southern China^[19]^ and 12.5/100 PY (95% CI: 9.1–15.7) in Chongqing in southwestern China.^[20]^ A multi-center prospective cohort study conducted showed moderate HIV incidence in second-line large cities, such as 5.3/100 PY in Kunming, 4.8/100 PY in Nanning, and 4.3/100 PY in Urumqi.^[8]^ It was suggested that HIV epidemic has been expanding in this population in both first-line and second-line cities across the country. HIV incidence among Chinese MSM is higher than that in other large cities in Asia and other continents, for example, 4.7/100 (95% CI: 4.0–5.4) PY in Bangkok, Thailand,^[21]^ 4.11/100 PY (95% CI: 2.8–6.0) in Chicago, USA,^[22]^ and 1.3/100 PY (95% CI: 1.0–1.6) in Melbourne, Australia.^[23]^ High HIV incidence as shown in this study, combined with high HIV prevalence in cross-sectional studies in Hangzhou,^[13–15]^ highlighted the emergency of developing targeted interventions for MSM.

The retention at 6 months in this study is only 26.2%, which is lower than 61% to 86% in other cohort studies among Chinese MSM, for example, cohort retention at 12-month ranging from (81%–86.8%).^[7–10]^ The reasons may include high proportion of migrant participants, lack of aggressive plan of follow-up, and lack of incentives for follow-up. Participants who reported being homosexual were less likely to be retained, as they might confront homosexuality-related stigma. Participants who were recruited via Internet were also less likely to be retained in cohort compared with those recruited via other methods. It is suggested that Internet is a useful venue for recruiting this hard-to-reach study population, but additional efforts are needed to retain the participants in prospective cohort studies or clinical trials. Previous studies have also shown that age and education affected cohort retention among MSM. Older MSM had higher cohort retention,^[24–26]^ older MSM might have more stable life in local area and pay more attention on their health, which might increase cohort retention.^[26]^ Higher level of education was associated with higher retention, as MSM with higher education might be less likely affected by social stigma and discrimination.^[27]^ It is a challenge to increase cohort retention in both research and public health intervention programs among this population.

The prevalence of syphilis in our study sample was 6.5%, lower than in other studies among Chinese MSM.^[27–29]^ Men who disclosed their sexual orientation had a lower risk of syphilis than those did not, as the former ones might have less self-denial and self-discrimination. Self-discrimination could lead to high-risk sexual behaviors.^[30,31]^ Multiple comprehensive HIV intervention programs have been implemented targeting Chinese MSM in recent years, including treatment of HIV/STIs and provision of pre-exposure prophylaxis,^[32–34]^ but these programs often lacked of psychological counseling on acceptance of their sexual identity among MSM.

Our study has limitations. First, non-random sampling methods were used for recruiting study participants, as random sampling is unlikely to recruit this hard-to-reach population. This non-random nature of sampling may lead to selection bias. Second, the high rate of loss to follow-up may lead to overestimation or underestimation of HIV incidence. Third, HIV infected participants were excluded at baseline investigation, this may lead to underestimation of syphilis prevalence.

In summary, this study revealed high HIV incidence among MSM in the second-line Chinese city Hangzhou. Effective interventions are needed to cover MSM living in both first-line megacities and second-line large- and middle-size cities in China.

## Author contributions

**Conceptualization:** Xiting Li, Qingchun Li, Han-Zhu Qian.

**Formal analysis**: Qingchun Li, Dai Fang, Han-Zhu Qian.

**Funding acquisition:** Xiting Li, Yan Luo.

**Investigation:** Qingchun Li, Xiting Li, Yan Luo, Xingliang Zhang, Xin Lv, Jie Jin, Ke Xu, Wenjie Luo.

**Laboratory tests:** Ke Xu, Wenjie Luo.

**Project administration:** Junfang Chen, Han-Zhu Qian.

**Supervision:** Xiting Li, Han-Zhu Qian.

**Writing – original draft:** Qingchun Li, Dai Fang.

**Writing – review & editing:** Han-Zhu Qian.

Qingchun Li orcid: 0000-0001-6823-5074.

Xiting Li orcid: 0000-0001-8149-0934.

Han-Zhu Qian orcid: 0000-0003-2785-2628.
